# Integrated environmental management and GPS-X modelling for current and future sustainable wastewater treatment: A case study from the Middle East

**DOI:** 10.1016/j.heliyon.2024.e34164

**Published:** 2024-07-05

**Authors:** Ayat Sami Odeibat, Reham Mohammad, Majed Abu-Zreig

**Affiliations:** a University of Debrecen, Faculty of Economic and Business, Karoly Ihring Doctoral School of Management and Business, H-4032 Debrecen, Böszörményi út 138, Hungary; b Jordan University of Science and Technology, Department of Civil Engineering. Irbid, Jordan

**Keywords:** Wastewater treatment plant efficiency, Environmental sustainability, Water resource management, Modelling and simulation, Technological innovations

## Abstract

In the context of today's rapidly changing environmental challenges, accurately predicting the performance and efficiency of environmental management strategies is crucial. Particularly in the Middle East, where research on wastewater treatment plants (WWTPs) is notably lacking, addressing this need is imperative. This study investigates the treatment efficiency of a wastewater treatment plant and proposes various techniques to enhance its performance. Employing a case study method, we utilise the GPS-X model to forecast the plant's performance under diverse scenarios, offering solutions for future challenges. The results reveal that the current plant layout operates efficiently, with removal efficiencies for Total Suspended Solids (TSS), Chemical Oxygen Demand (COD), and Biochemical Oxygen Demand (BOD) at 98.3 %, 95.1 %, and 96.1 %, respectively. The outlet Dissolved Oxygen (DO) of 1.9 mg/L meets local wastewater reuse standards. Furthermore, the GPS-X model forecasts the plant's performance under different scenarios, suggesting the feasibility of a new layout within 20–25 years and the need for additional units after 40 years. As inflow approaches maximum design capacity, simulation results underscore the importance of utilising the full plant design and expanding it for optimal operation over 60 years. This research provides critical insights for improving WWTP performance and emphasizes the significance of strategic planning in addressing long-term environmental management challenges. Moreover, this study represents a pioneering effort in addressing critical water scarcity challenges in Jordan by exploring the potential of treated wastewater (TWW) as a sustainable solution, thus contributing to the advancement of environmental management practices in the region.

## 1 Introduction

Environmental challenges have recently become a global concern. The exceptional magnitude of today's environmental challenges is indisputable within the realm of serious scientific discourse. The understanding of the enormity of these challenges continues to expand [1]. These challenges encompass climate change, waste management, water management, pollution, deforestation, and degradation, all of which are interconnected and complex problems that necessitate multifaceted approaches and dynamic solutions. Consequently, it is critical to consider different sorts and sources of knowledge [2] to mitigate their impacts and assure a sustainable future for the earth [3,4].

This research investigates water management; specifically wastewater treatment and water sources. Water management efficiency can be measured by its environmental impact. Water efficiency, wastewater treatment and reuse, and avoiding negative environmental impacts are all good indicators of good and sustainable management [5,6]. Water resource mismanagement, environmental and water pollution, over-exploitation of water resources, violations of sustainability regulations, and failure to respect intergenerational equity all point to inefficient water resource management [6]. In general, the water situation in the Middle East is challenging, in a region in which climate change is creating water insecurity with a projected deficit of 2 billion cubic meters by 2050 [7]. In addition, the region is consistently grappling with depleted water resources due to poor management and resource degradation to the extent that some water sources are unsuitable for human consumption. The rapidly increasing population, food requirements, and urbanisation, are further intensifying the strain on these diminishing resources. Meanwhile, global warming is exacerbating persistent and severe droughts, leading to increased competition for water resources across various sectors and even among different states, as well as contributing to agricultural difficulties [8]. Therefore, this presents a significant challenge for both the population and the economy of the region [7].

Jordan is a country that has suffered from water shortages for many years and is considered one of the most water scarce countries in the world [9,10]. The renewable water supply in the country covers only about a half of the water demand [11]. Additionally, groundwater in Jordan is being depleted faster than it is being replaced [12]. The population growth and the influx of refugees from conflict zones put a further strain on water resources, alongside changes in the amount and quality of rainfall, all of which add to the stress [12]. In addition, various sectors are experiencing significant development, including a strong manufacturing sector, an exceptional level of health care and education, and a strong information technology sector, which increases the demand for water resources, therefore making it imperative to explore effective solutions for securing new sources of water. These sources should be applicable in industry, agriculture, and other fields [7,13,14]. In response to these challenges, in this section, we will explore potential solutions and strategies for environmental management. One of the most promising strategies for extending and conserving available water sources is the use of treated wastewater (TWW). TWW is considered one of the most crucial and effective solutions to address the water shortage in Jordan [14,15]. It is defined as the process of removing pollutants and impurities (reclamation) from wastewater and changing its properties to make it more acceptable in the environment or converting it into water that can be used in multiple applications [[16], [17], [18]]. These treatment processes occur in wastewater treatment plants (WWT), also known as water resource recovery facilities (WRRF) or sewage treatment plants (STP). In these facilities, pollutants present in the wastewater are reduced, diverted, or broken down during the treatment process.

The treated wastewater (TWW) can be used in various sectors, including agriculture, landscape irrigation, artificial recharge, and industries [19,20]. For instance, the use of TWW is one of the most effective ways to address water shortages and nutrient requirements in agricultural systems [14]. Although the use of TWW can alleviate water shortages, it must be applied in a controlled environment to reduce the health risks caused by pathogenic and toxic pollution of agricultural products, soils, surface, and groundwater. The greatest challenge is to maximise the benefits of TWW as a resource while minimising its negative impact on human health [21]. Jordan prioritises reuse activities to ensure the highest possible impact and efficiency. To achieve the greatest efficiency, it is important to conduct relevant simulations and forecasts for WWTP outcomes. Given the limited research and investigation into wastewater treatment plants (WWTPs) and the utilisation of the GPS-X model within the Middle Eastern context, especially in Jordan, there is a growing necessity for further exploration in this area.

Therefore, this study presents several novel contributions to the field of wastewater treatment in Jordan. Firstly, it is the first known research endeavour in the country to utilise the GPS-X model for optimising wastewater treatment processes. By employing this advanced modelling tool, the study pioneers a novel approach to enhance the efficiency and performance of wastewater treatment plants. This unique application of the GPS-X model in the Jordanian context not only fills a significant research gap but also establishes a valuable reference for future researchers and practitioners in the field. Additionally, the study addresses critical water scarcity challenges in Jordan by exploring the potential of treated wastewater (TWW) as a sustainable solution. By providing insights into the utilisation of TWW and proposing optimisation strategies using the GPS-X model, the research contributes to the advancement of environmental management practices in the region. Overall, the novelty of this study lies in its innovative use of the GPS-X model for wastewater treatment optimisation and its potential to serve as a foundational resource for future studies in Jordan and beyond.

The modelling and simulation system GPS-X is a contemporary commercial computer program that accurately simulates the operation of wastewater treatment plants. It aids in the effective management of these plants, ensuring they operate under optimal conditions to achieve desirable results and specifications [22]. In the Middle East, GPS-X model analyses have been conducted to optimise wastewater treatment processes. For example, a GPS-X simulator was used in these studies to model and simulate wastewater treatment plants, such as the Karbala wastewater treatment plant in Iraq [23]. The application of advanced simulation software is beneficial in cost savings and decision-making processes by reliably assessing the TWW quality in different circumstances [24]. The research has focused on improving the denitrification process, lowering phosphate concentrations, and enhancing the overall performance of WWTPs [25]. Previous studies in Jordan have not specifically referenced the use of GPS-X model investigations aimed at enhancing wastewater treatment procedures. Therefore, this study provides insightful information about the use of GPS-X modelling in optimising wastewater treatment processes in the country.

Ultimately, the main goal of this study is to examine the treatment efficiency of the WWTP and propose various techniques and scenarios to improve its efficiency using the GPS-X model. Furthermore, the research seeks to achieve several specific objectives, which include assessing the removal rates of key pollutants such as BOD, COD, and TSS, while simultaneously evaluating the performance of treatment units. Additionally, it aims to ensure that the quality of effluent meets regulatory standards and to validate the predictive capabilities of the GPS-X model by comparing simulated results with empirical data. Another objective is to forecast the performance of the plant under varying conditions, including changes in influent characteristics, population growth, wastewater inflow volume, and regulatory requirements. Furthermore, the research seeks to develop predictive models that can forecast modifications in plant performance over extended time horizons. Finally, it aims to evaluate the trade-offs between different treatment scenarios, considering their environmental, economic, and social implications. As a result, the following research questions were created: What is the current efficiency of the MWWTP? To what extent does the GPS-X model accurately reflect the reality of the Al-Marad WWTP? How can the best strategies for optimising long-term WWTP performance be predicted? What is the optimal scenario for achieving the highest removal efficiency at the lowest cost for the WWTP?

## 2 Materials and methods

### 2.1 The Al-marad wastewater treatment plant as one of the pioneer stations in the Middle East

To achieve the research objectives, this study establishes criteria for selecting WWTPs in Jordan, emphasising the importance of comprehensively understanding the situational context and unique characteristics of these facilities. In Jordan, wastewater is categorised into domestic and industrial types [26]. The treated wastewater is primarily used for restricted agriculture irrigation, and any excess treated water is discharged by gravity into valleys and Dams. The selection of the Al-Marad wastewater treatment plant as the focus of this study was driven by several factors. Firstly, it stands out as a modern plant equipped with advanced technological systems, having commenced operations in 2011. Surprisingly, despite its technologically advanced nature and operational history, no prior studies have been conducted on this facility, making it an intriguing subject for investigation. Moreover, its unique location in a region of the country where agriculture is very important further underscores its relevance for researchers. Notably, the Al-Marad wastewater treatment plant was constructed using the GPS-X model, highlighting its innovative approach to wastewater management. This utilisation of advanced modelling technology aimed to optimise the plant's performance, enhance the effectiveness of its treatment units, and prolong its operational lifespan. By conducting research on this specific facility, we sought not only to ensure its continued efficiency and functionality but also to establish it as a benchmark for other wastewater treatment plants across the kingdom.

The Al-Marad WWTP utilises an activated sludge system with extended aeration, where microorganisms in the wastewater are preserved in a suitable environment in the aeration basins to facilitate oxygen supply [27]. These microorganisms consume organic materials, multiply, and form activated sludge clusters, which settle in the final sedimentation basins. Excess sludge is recycled or transferred to drying beds and dewatering machines. Maintaining treatment efficiency requires balancing the BOD of incoming wastewater with the suspended organic matter concentration, governed by standards such as the F/M ratio (0.5–1), sludge age (13–20 days), and daily SVI stability tests.

This paper utilises a case study approach to fulfil the research objectives. An initial step involves evaluating the treatment plant by analysing the type, volume, and characteristics of inflow wastewater. The examination further entails assessing the current treatment units within the plant, and evaluating their suitability and capacity to manage the inflow volume. The methodology involves several key steps: weekly sampling for six months, adhering to Jordanian standards, to analyse and collect data at the plant's entry point, and measuring parameters such as inflow, BOD, COD, TDS, TSS, pH, and temperature. Subsequently, the evaluation of WWTP efficiency is conducted by comparing the concentration of these attributes upon entry and exit from the facility. The process also includes preparing and constructing a GPS-X model to mirror the WWTP's real conditions. Utilising the simulation feature in the GPS-X Model, scenarios are tested by manipulating treatment process parameters to determine the most efficient scenario that optimises removal efficiency at the lowest cost for the WWTP. The following formulas were used to calculate the removal efficiency law and the population growth.

(1) $$\text{Removal}\;\text{Efficiency}=\frac{Cin-Cout}{Cin}*100\%$$

where: C _in_ is the inflow characteristics, and C _out_ is the outflow characteristics.

(2) $$\text{Pt}=\text{Po}\;{\left(1+i\right)}^{t}$$

where P_t_ is the number of populations after t of time, P_o_ is the current population number, i is the annual growth rate and t is the time in years.

The plant's operational system encompasses various critical components observed on-site: screens, essential for eliminating solid materials obstructing the purification process, are currently out of service and in need of maintenance. The grit chamber, crucial for removing sand and gravel to prevent equipment erosion, is presently inactive. Both anaerobic tanks designated for phosphorous removal and one aeration tank are out of service. Only one secondary sedimentation tank is operational, while sand filters, responsible for removing sediments, are inactive. The chlorine disinfection system operates intermittently, and only one of two sludge thickener tanks is in service. Seven drying beds, cost-effective and easy to maintain, are in good condition. Of the sludge dewatering units, one requires maintenance, and the other remains in service. Lastly, the equalisation tank, used during peak inflow periods, is currently non-operational.

### 2.2 Modelling and simulation

Modelling and simulation are defined as the use of physical, mathematical, logical, or other types of models in simulations to create a computerised representation of a real situation. This allows us to obtain the best and most appropriate solution(s) and decision(s) for a real world situation. Additionally, it can explore the problem under different scenarios, aiding in comparisons and the selection of the correct decision. In the field of engineering, simulations and modelling find wide application across various disciplines. Simulations provide approximations and simplifications of the real-world scenario, enabling the addition and modification of inputs to any system. This, in turn, facilitates error monitoring, identification of the causes of problems, and the development of optimal solutions [28].

#### 2.2.1 Description of GPS-X

GPS-X is a design program used for the simulation and design of wastewater treatment plants. The GPS-X software was developed by the Hydromantis Company to provide a clear understanding of wastewater treatment plant performance and assist in predicting the plant's future. The program library contains numerous processes for treating wastewater and solid materials, along with a comprehensive range of biological treatment processes that address nitrogen, phosphorus, carbon, and pH levels. In terms of design, GPS-X is utilised to assess the impact of increased biological loads on treatment plant facilities and to operate the plant under various scenarios, thus saving costs and effort. Additionally, GPS-X examines the effects of internal recycling rates, anoxic zones, and anaerobic zones on processes such as nitrification, denitrification, and overall treatment effectiveness [29]. GPS-X can be summarised as follows.

• Designing unit processes: primary treatment, secondary treatment, and biosolids handling.
• Minimising operational costs while meeting effluent quality requirements.
• Predicting the effects of taking one of the unit processes offline for maintenance.
• Ensuring a swift recovery from plant upsets.
• Accurately evaluating process control improvements.
• Providing operator training by illustrating the impact of operating decisions on plant performance.

One of the advanced features of GPS-X is the drag, drop, and link capability. With this feature, designers and analysts can combine processing units, and add, or delete the connections between them to create different scenarios. It is also known for its efficiency in entering information for the units used. Additionally, GPS-X can be integrated with MatLab. The software is further distinguished by the presence of its optimiser [22,29].

The utilisation of GPS-X in the modelling process entails employing simulation software to analyse and enhance the efficiency of water treatment facilities. GPS-X functions as a specialised software suite tailored for modelling and simulating wastewater treatment processes. Drawing from the historical performance data of the plant, GPS-X constructs dynamic models capable of simulating various scenarios and refining the treatment process [30]. By converting graphical representations of processes into material balance equations, the software facilitates the kinetic depiction of treatment processes and the examination of crucial parameters [31]. Moreover, GPS-X serves for the design and simulation of sewage treatment plants, providing calculations and specifications for individual unit operations [32]. Furthermore, GPS-X aids in the calibration of mathematical models for activated sludge processes, enabling the analysis of plant performance and capacity assessment. In summary, GPS-X emerges as a potent instrument for both modelling and optimising water treatment processes.

The GPS-X modelling process encompasses several key steps. It commences with data collection, where pertinent information regarding the wastewater treatment plant (WWTP), including influent characteristics and effluent quality, is gathered. Following this, a digital representation of the WWTP is established through the model setup, detailing its treatment units, pipelines, and processes. Calibration then ensues, during which the model is fine-tuned to accurately mirror the behaviour of the actual plant, involving adjustments to parameters and settings based on observed data. Subsequently, simulation is conducted to predict the WWTP's performance under various scenarios, potentially incorporating changes in influent characteristics or the addition of new treatment units. An analysis phase follows, assessing the WWTP's performance in order to pinpoint areas for improvement, such as comparing simulated effluent quality with regulatory standards and optimising operational strategies. Optimisation strategies are then developed to enhance the efficiency and effectiveness of the WWTP, which may involve adjustments to operational parameters or the implementation of new technologies. Finally, the model results undergo validation against real-world data to ensure their accuracy, affirming the reliability of the model and the efficacy of the proposed optimisation strategies [[30], [31], [32]].

#### 2.2.2 Influent characteristics

The practical aspect of this study relies on direct observation and the measurement of various wastewater parameters that enter the treatment plant over six months. Readings were collected twice a week, with samples obtained in the morning from the plant's entrance and subsequently measured in the laboratory. The parameters measured include inflow, BOD, COD, TDS, TSS, pH, and temperature. Table 1 shows the average influent characteristics over the six month period, including the standard deviation (see Table 2).

**Table 1:** Influent readings during the six-month study period.

Influent parameter	Readings average/month							
	July	August	September	October	November	December	6-month average	Standard Deviation
Inflow m^3^/day	3820	3950	3760	3804	4090	899.3	4000	1118.059
BOD mg/L	1050.9	891.69	1053.4	1029.4	960.4	899.3	980.8	67.75657
COD mg/L	1449.6	1116.3	1224.1	1336.4	1259.9	1192.4	1263.1	106.642
TSS mg/L	1056.7	900.4	1004.9	1001.1	1019.9	997.8	996.8	47.4336
TDS mg/L	2423.3	1774.4	2023.6	1919.8	2038.4	2018	2032.91	196.7929
DO mg/L	1.1	0.9	1.2	1.3	1.5	1.6	1.3	0.235702
pH	7.4	7.4	7.6	7.6	7.4	7.3	7.5	0.111803
Temperature C^o^	19.4	19.2	18	18.3	16.2	16.1	17.9	1.305969

This study aims to assess the performance of the Al-Marad WWTP, which necessitates the examination of its effluent and a comparison of its characteristics with the Jordanian standards. Hence, it was imperative to measure various parameters of the effluent and compare them with those of the influent. The following table shows the average effluent characteristics throughout the study period, in addition to calculating the standard deviation.

**Table 2:** Effluent readings during the six-month study period.

Effluent parameter	Readings average/month							
	July	August	September	October	November	December	6-month average	Standard Deviation
BOD mg/L	50.7	46.4	34.3	24.9	21.8	17.9	32.6	12.33514
COD mg/L	72.7	59.3	50.4	44.5	43.5	45.3	52.6	10.45186
TSS mg/L	21.4	17.1	13.3	17.6	14.9	14.1	16.4	2.710474
TDS mg/L	997	964.1	958.3	964.9	970.3	970.1	970.8	12.40382
DO mg/L	1.63	1.66	1.95	2.1	2.07	2.1	1.91	0.199951
pH	7.5	7.3	7.2	7.23	7.3	6.9	7.2	0.178924
Temperature C^o^	26.2	28.7	28.7	28.4	28.3	28.2	28	0.862973

This research built the model to evaluate and assess the WWTP's current situation and mirror the WWTP's real conditions in order to predict its future outcomes and sustainability. The results section will present the current and future scenarios.

## 3 Results

The evaluation process relies on a comparison of the inputs and outputs of the treatment plant, ensuring that the treated wastewater adheres to the specified local standards. In this section of the study, the efficiency of the Al-Marad WWTP will be quantified by assessing the quality of the effluent water and subsequently comparing it with the recommended specifications. The results were categorised into two primary sections: an evaluation of the Al-Marad Wastewater Treatment Plant based on the influent and effluent readings, and an assessment derived from a simulation of the plant's performance using the GPS-X Model.

### 3.1 Evaluation of Al-marad WWTP performance/field results

The efficiency of the Al-Marad WWTP will be calculated and its performance will be evaluated based on the laboratory test results. The removal efficiency of the plant will be calculated based on the removal effectiveness law. The general efficiency law, which theoretically calculates the difference between the inflow and outflow of the plant, shows the changes made by the treatment units. This is shown in Table 3 which displays the six-month average of the BOD, COD, TDS, TSS, pH, and temperature readings that were taken at both the inlet and the outlet of the plant.

**Table 3:** The 6-month average of the BOD, COD, TDS, TSS, pH and temperature readings that were taken at both the inlet and the outlet of the plant, and the efficiency removal % of the Al-Marad WWTP.

Parameters	Average readings during the study period			
	Average inlet reading	Average outlet reading	Reduction	Removal Efficiency %
BOD mg/L	984.4	36.9	947.4	96.2 %
COD mg/L	1198.8	50.6	1148.1	95.2 %
TSS mg/L	996.9	16.4	980.4	98.3 %
TDS mg/L	2032.9	970.8	1062.1	51.3 %

The outlet DO concentration ranged from 1 to 2.2 mg/L, while the pH and temperature adhered to the required standards, being 7.5 and 25–35 °C, respectively. Laboratory tests indicated that the treated wastewater from the Al-Marad WWTP is suitable for irrigating fruit trees and green areas. Additionally, it can be discharged into valleys and streams or utilised to recharge unused groundwater wells not designated for drinking purposes.

### 3.2 Evaluation of Al-marad WWTP performance/modelling results

**Fig. 1:**
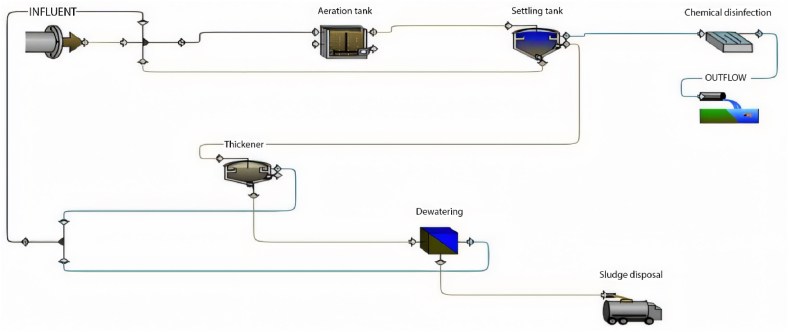
Base model of Al-Marad WWTP at the present time.

GPS-X is an advanced software designed for simulating WWTP performance, offering numerous options and units commonly utilised in wastewater treatment plants. The base model is established and calibrated using data provided from the plant, encompassing design criteria, flow rate characteristics of the influent, and operational data. The base model comprises solely the units currently in operation at the plant. Fig. 1 illustrates the base model of the Al-Marad WWTP as of the current time.

To calibrate the model, the physical and chemical characteristics of the inlet wastewater were considered. Inlet characteristics were input based on laboratory findings: COD was set at 1300 mg/L, total phosphorous at 12 mg/L, total Kjeldahl nitrogen (TKN) at 57 mg/L, ammonia nitrogen at 28 mg/L, and orthophosphate at 9 mg/L, with an average inflow of 4000 m^3^/day. Additionally, the influent fraction was adjusted from default values using data extracted from the plant's manual. Filtered BOD, filtered COD, and fluctuate COD were employed to compute these fractions. Table 4 provides a comparison between the actual and default values of fractions utilised in GPS-X.

**Table 4:** Comparison of actual and default values for the fractions used by the GPS-X model.

Fraction	Definition	Used value	GPS-X Default value
Frsi	Soluble inert fraction of total COD	0.03	0.05
Frslf	VFS fraction of total COD	0	0
Frsf	Fermentable biodegradable fraction of total COD	0.2	0.2
Frxi	Particulate inert fraction of total COD	0.1	0.13
Frxs	No slowly biodegradable substrate	0	0
Insi	N content of soluble inert material	0.05	0.05
Inxi	N content of particulate inert material	0.04	0.05
Frsnh	Ammonium fraction of soluble TKN	0.9	0.9
Ipsi	P content of soluble inert material	0.01	0.01
ipxi	P content of particulate inert material	0.01	0.01
Frss	Readily biodegradable fraction of total COD	0.3	0.2
Frcas	Acetate fraction of total COD	0	0
Frcsol	Colloidal fraction of slowly biodegradable COD	0.13	0.15
SVI	Solid Volume Index	150	159.7

A simulation of the Al-Marad WWTP was created based on the data entered into the model. The results of 30-day of simulation using the GPS-X model are shown in Table 5.

**Table 5:** Effluent simulated results.

Parameter	Unit	Simulation value
Flow	m^3^/d	3990.7
TSS	mg/L	16.1
BOD_5_	mg/L	30
COD	mg/L	7.8
pH	–	7
DO	mgO_2_/L	1.5

**Fig. 2:**
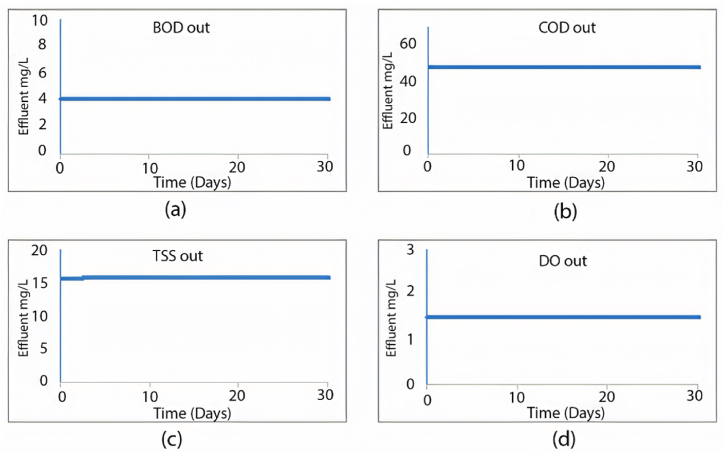
Effluent simulated results at the current layout for (a) BOD mg/L, (b) COD mg/L, (c) TSS mg/L, and (d) DO mg/L.

When calibrating the GPS-X model, it efficiently mirrored the treatment plant's effectiveness. The removal efficiencies obtained for COD, BOD, and TSS were 96.1 %, 69.9 %, and 98.5 %, respectively. Comparatively, the treatment plant achieved removal efficiencies of 95.2 % for COD, 96.2 % for BOD, and 98.3 % for TSS. Fig. 2 illustrates the 30-day simulation results of the current plant's effluent COD, TSS, and BOD.

**Fig. 3:**
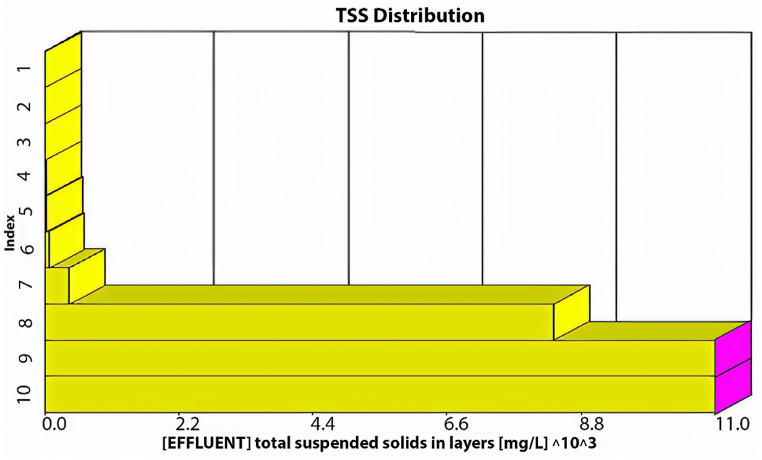
TSS distribution through the sedimentation tank's layers using the current layout.

The GPS-X model offers an option that allows the user to determine the amount of sediment in the sedimentation tanks. These tanks are divided into ten equally thick layers each showing the sediment concentration. Fig. 3 illustrates the distribution of TSS in these layers within the sedimentation tank. The illustration indicates that the first six layers effectively maintain acceptable TSS effluent levels, while sediment accumulation significantly affects the remaining layers. Consequently, TSS effluent from the first layer meets standards.

### 3.3 Evaluation of Al-marad WWTP performance using the GPS-X model with subsequent future scenarios

This section presents the performance analysis of the Al-Marad WWTP under different inflow rates. Through modelling, we will showcase the operational schedule for treatment units over time as the plant approaches its maximum design capacity of 10,000 m^3^/d. Additionally, we will simulate potential future scenarios in which influent rates exceed 10,000 m^3^/d, forecasting the expected influent rate at which the Al-Marad WWTP could potentially fail, and the corresponding date.

#### 3.3.1 The base model performance at the maximum inflow design (10,000 m^3^/day)

**Fig. 4:**
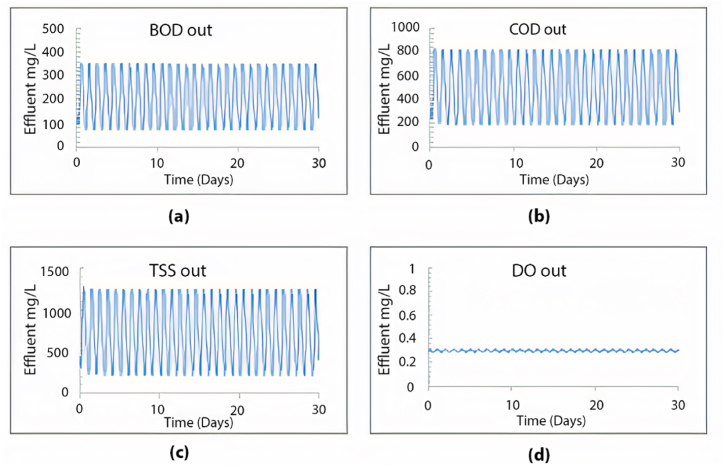
Effluent simulated results for (a) BOD mg/L, (b) COD mg/L, (c) TSS mg/L and (d) DO mg/L with maximum design inflow conditions.

The plant is designed to handle 10,000 m^3^/day. In this section, the current plant layout (Fig. 2) will be assessed under the operating conditions equivalent to the design inflow of 10,000 m^3^/day. The results obtained from a 30-day simulation are summarised in Fig. 4.

**Fig. 5:**
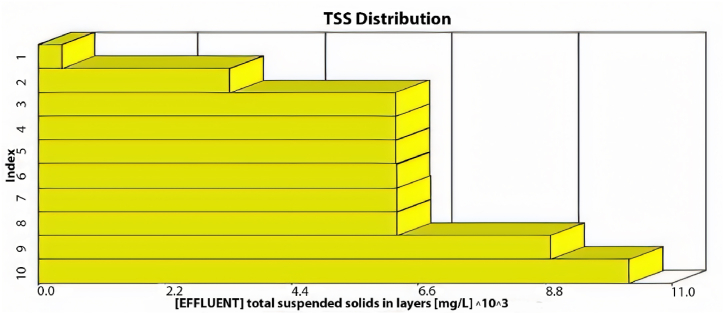
TSS distribution through the sedimentation tank's layers at the maximum inflow conditions using the current layout.

As illustrated in Fig. 4, the average effluent COD was 500 mg/L, the average effluent BOD was 200 mg/L, and the effluent TSS was 720 mg/L, with an average effluent DO of 0.3 mg/L. Our analysis indicates that the existing plant layout fails to meet the design standards at maximum capacity, as all effluent characteristics exceed acceptable limits. Moreover, under maximum inflow conditions, the average TSS effluent from the initial sedimentation tank layer would reach an unacceptable 700 mg/L, as depicted in Fig. 5.

#### 3.3.2 Performance evaluation beyond 5 years

In this section, the impact of the inflow volume on the efficiency of the Al-Marad WWTP will be investigated using the GPS-X model. The inflow volume calculation relied on an exponential model predicting population growth. This model estimates population growth over a specific period, assuming a constant growth rate, based on the population formula [33].

The number of homes connected to the sewage network in 2020 is approximately 5750 houses, accommodating roughly 28,800 individuals. Based on the Jordanian growth rate of 2.3 % and this correlation, the estimated population by 2025 (in five years) will be around 32,268 persons.

**Fig. 6:**
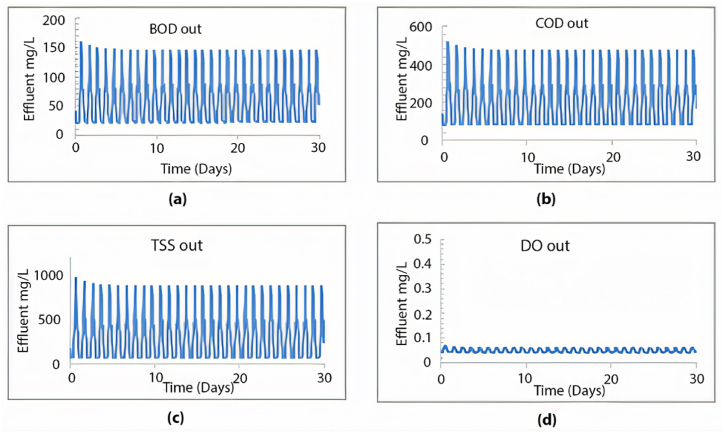
Effluent simulated results beyond 5 years using the current layout for (a) BOD mg/L, (b) COD mg/L, (c) TSS mg/L and (d) DO mg/L.

This scenario was replicated using the GPS-X model to analyse the impact of increased inflow volume on the efficiency of the wastewater treatment plant over a period of more than five years, using the current layout. After a 30-day simulation, the effluent levels of TSS, COD, BOD, and DO are summarised in Fig. 6.

**Fig. 7:**
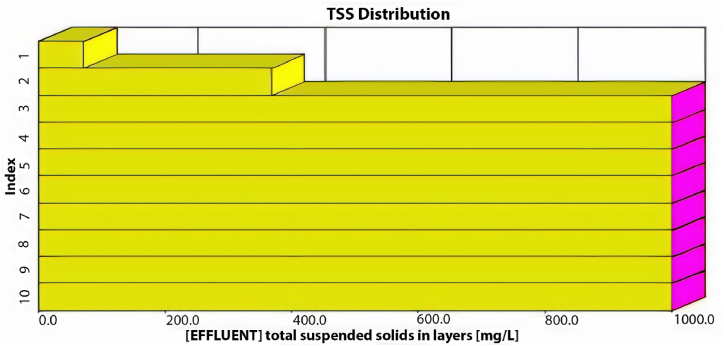
TSS distribution within the sedimentation tank's layers beyond 5 years.

As depicted in Fig. 6, the current configuration of the treatment plant cannot manage 4481.6 m^3^/day, resulting in unsatisfactory treatment efficiency. The average effluent levels were as follows: TSS at 290 mg/L, COD at 190 mg/L, BOD at 57 mg/L, and DO at 0.05 mg/L. None of these effluent characteristics meet the required standards for treated wastewater. Fig. 7 shows a high concentration of TSS in all sedimentation tank layers, with the effluent TSS from the first layer averaging 89 mg/L, an unacceptable level.

**Fig. 8:**
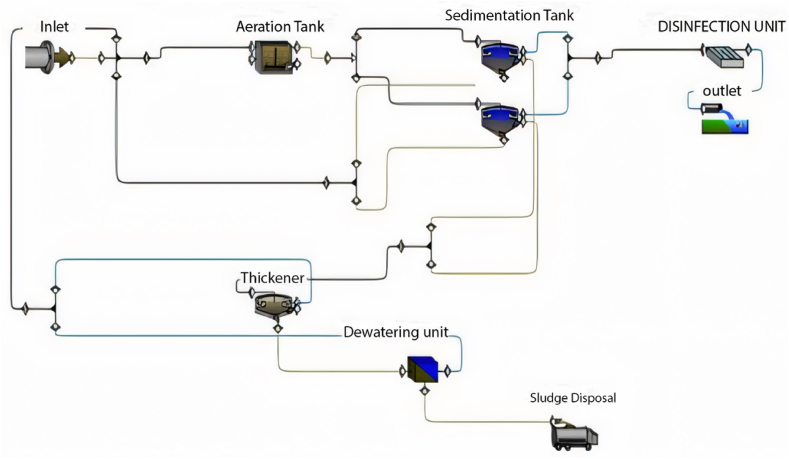
The proposed layout beyond 5 years.

Based on the simulation results in this scenario, it is evident that the current plant layout is incapable of managing an inflow of 4500 m^3^/day. Consequently, the current layout would not be sustainable after 5 years. Therefore, our recommendation is to activate the second sedimentation tank, as depicted in Fig. 8, to maintain treatment efficiency within acceptable limits.

The GPS-X model was subsequently employed to simulate the MWWTP with the second sedimentation tank in operation. Fig. 10 displays the outcomes for effluent TSS, COD, BOD, and DO after a 30-day simulation.

**Fig. 9:**
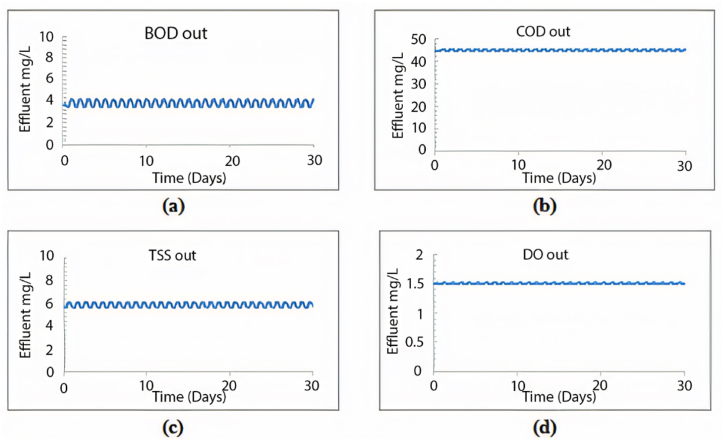
Effluent simulated results beyond 5 years using the proposed layout for (a) BOD mg/L, (b) COD mg/L, (c) TSS mg/L and (d) DO mg/L.

**Fig. 10:**
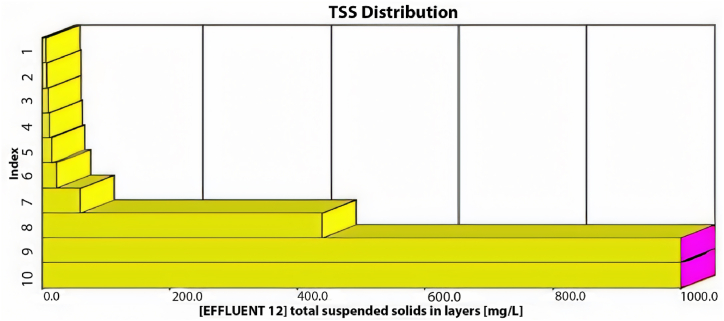
TSS distribution through the layers of both sedimentation tanks using the proposed layout beyond 5 years.

Observing Fig. 9, it is evident that the plant's treatment efficiency meets the standards, as all effluent concentrations for COD, BOD, and TSS remained below the specified limits. Adding a second sedimentation tank led to significant efficiency improvements, achieving a 99.6 % removal rate for TSS, 96.7 % for COD, and 99.7 % for BOD, maintaining an average effluent DO value of 1.51 mg/L. Additionally, this modified layout effectively resolves the sedimentation issue within the tank by distributing the inflow equally between both tanks, employing an equal split fraction of 0.5. As depicted in Fig. 10, the first seven layers yield a TSS effluent that complies with acceptable limits.

#### 3.3.3 Performance evaluation beyond 10–20 years

**Fig. 11:**
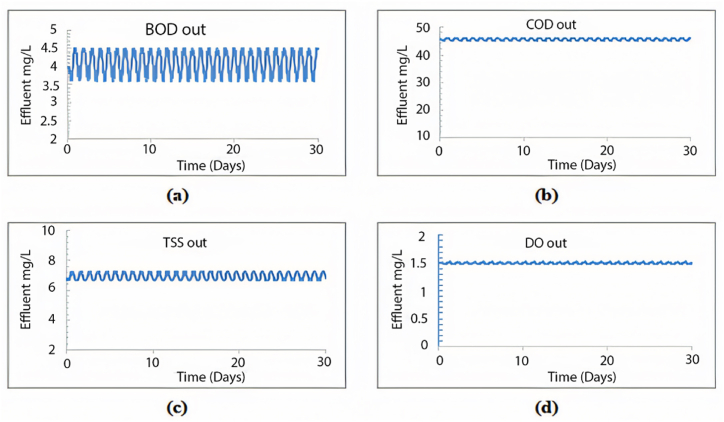
Effluent simulated results beyond 10 years using the proposed layout for (a) BOD mg/L, (b) COD mg/L, (c) TSS mg/L and (d) DO mg/L.

We simulated the performance of the Al-Marad WWTP at 5-year intervals until the treatment efficiency fell below the effluent standards. After 10 years, the plant is anticipated to cater to approximately 36,153 individuals, receiving an inflow of 5000 m^3^/day. We assessed the previous layout (depicted in Fig. 9) under the anticipated flow conditions for the next 10 years. The simulation results and the plant's effluent values for TSS, COD, BOD, and DO are presented in Fig. 11.

**Fig. 12:**
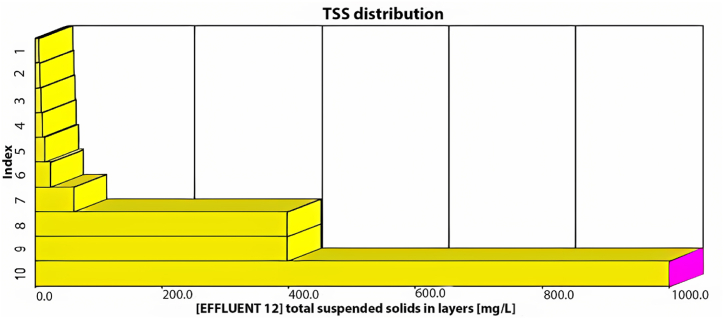
TSS distribution through the layers of both sedimentation tanks using the proposed layout beyond 10 years.

After a 30-day simulation, we determined that this layout is suitable for the next ten years. The concentrations of TSS, BOD, and COD do not exceed 50 mg/L, while DO remains under 1.5 mg/L, all in accordance with the relevant standards. The removal rates are 99.4 % for TSS, 96.5 % for COD, and 99.6 % for BOD. Furthermore, with this layout, both sedimentation tanks perform adequately, with the first six layers effectively maintaining TSS effluents below 50 mg/L, as depicted in Fig. 12.

**Fig. 13:**
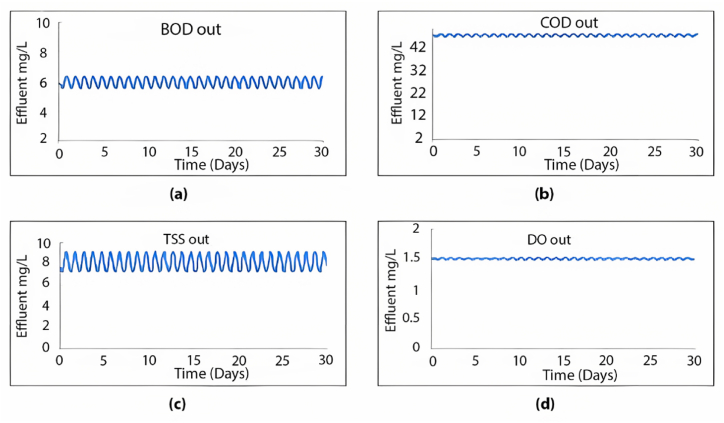
Effluent simulated results beyond 20 years using the proposed layout for (a) BOD mg/L, (b) COD mg/L, (c) TSS mg/L and (d) DO mg/L.

The same layout (Fig. 8) was examined for the 15- and 20-year periods, anticipating inflows of 5600 m^3^/day and 6300 m^3^/day, respectively. Fig. 13 displays the simulation outcomes after 20 years.

**Fig. 14:**
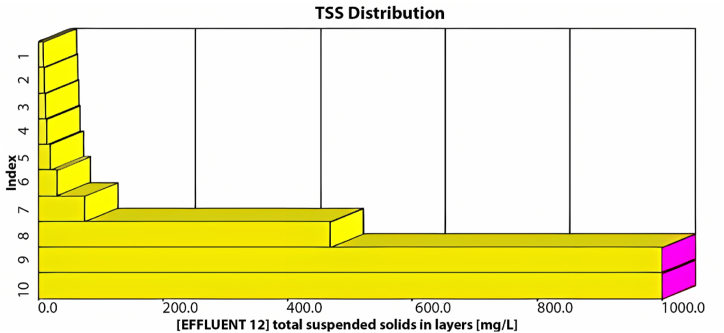
TSS distribution through the layers of both sedimentation tanks using the proposed layout beyond 20 years.

Based on Fig. 13, it is evident that the layout utilised after 5 and 10 years can also be effectively employed after 15 and 20 years. The plant's treatment efficiency remains acceptable, with all effluent concentrations for COD, BOD, and TSS well below the specified limits. The removal rates for TSS, COD, and BOD are 98.5 %, 96.4 %, and 98.5 %, respectively. Moreover, the sedimentation tanks effectively manage sediment accumulation, and the effluent TSS within the required limit is observed in the first six layers, as depicted in Fig. 14.

#### 3.3.4 Performance evaluation beyond 25–35 years

**Fig. 15:**
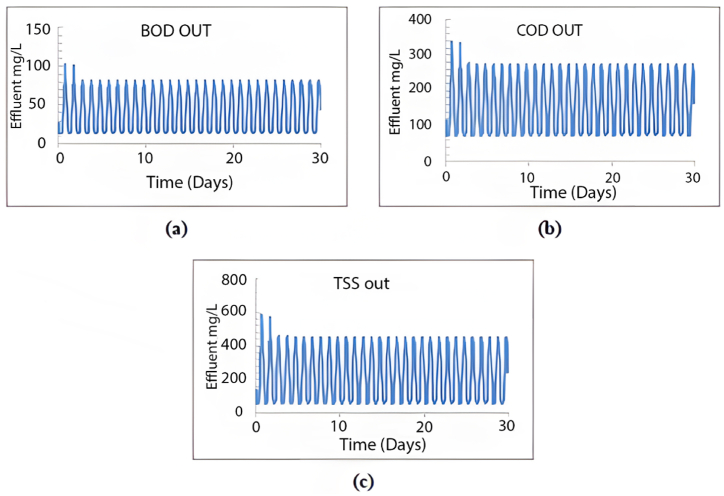
Effluent simulated results beyond 25 years for (a) BOD mg/L, (b) COD mg/L and (c) TSS mg/L.

In this scenario, the plant's performance will be tested over 25 years, serving 5000 people with an expected inflow rate of 7000 m^3^/day of domestic wastewater. Initially, we will assess the performance using the previous layout (5, 10, 15, and 20 years' layout, as illustrated in Fig. 8). Employing the GPS-X model, we simulated the plant's performance over a 30-day period. The results of this simulation are depicted in Fig. 15.

From Fig. 15, it is evident that the plant's effluents do not meet the permissible standards. The average effluent COD reached 160 mg/L, while the average value of BOD was 44 mg/L, and DO values were below 0.1 mg/L. Additionally, the sediments exceeded 10570.43 mg/L in each of the last six layers, with the first and second layers producing a TSS effluent with an average of 230 mg/L.

**Fig. 16:**
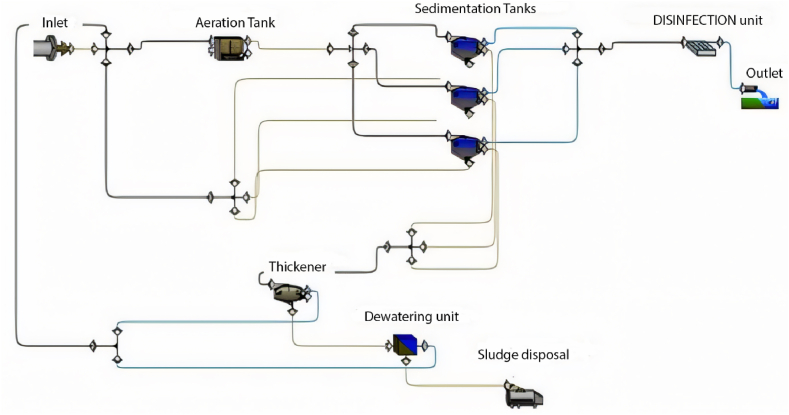
The proposed layout beyond 25 years.

The previous simulation results indicate that the current layout cannot effectively treat an inflow of 7000 m^3^/day. This suggests that the previously successful layouts used after 5, 10, 15, and 20 years are unsuitable at the 25-year mark. Therefore, it is necessary to modify the layout and incorporate the operation of the third sedimentation tank using equal split fractions, as illustrated in Fig. 16.

**Fig. 17:**
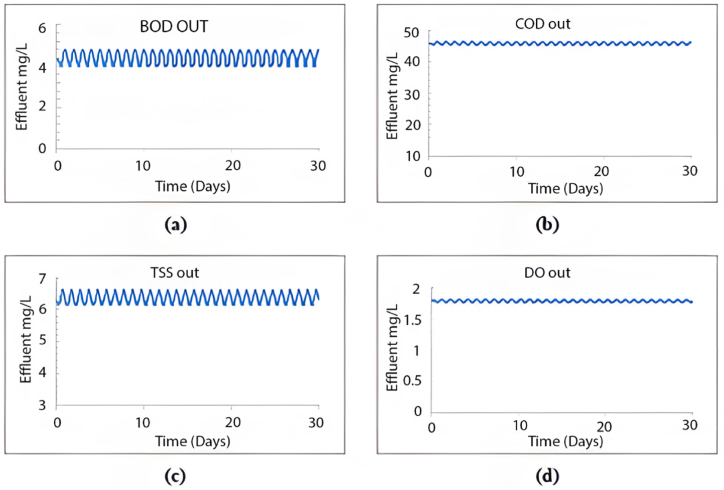
Effluent simulated results for the proposed layout (a) BOD mg/L, (b) COD mg/L, (c) TSS mg/L and (d) DO mg/L beyond 25 years.

The GPS-X model was used to simulate MWWTP performance with the third sedimentation tank in operation. Fig. 17 presents the results of effluent TSS, COD, BOD, and DO after a 30-day simulation.

**Fig. 18:**
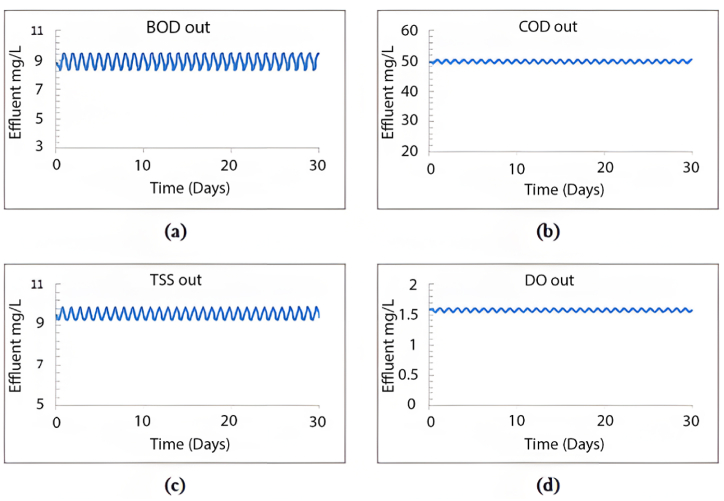
Effluent simulated results for the proposed layout (a) BOD mg/L, (b) COD mg/L, (c) TSS mg/L and (d) DO mg/L beyond 35 years.

Operating the third sedimentation tank produces good effluent results, with a TSS removal efficiency of 99.4 %, a COD removal efficiency of 96.5 %, a BOD removal efficiency of 99.6 %, and an average effluent DO of 1.79 mg/L, as shown in Fig. 17. Additionally, the first seven layers are in good condition, producing a TSS effluent of less than 50 mg/L. Following the simulation method used for every 5 years, a 30-day simulation was conducted to test the plant's efficiency beyond 35 years. The same layout adopted for beyond 25 years (Fig. 16) was tested for an inflow of 8800 m^3^/day. Fig. 18 presents the effluent COD, TSS, BOD, and DO resulting from using the same layout.

**Fig. 19:**
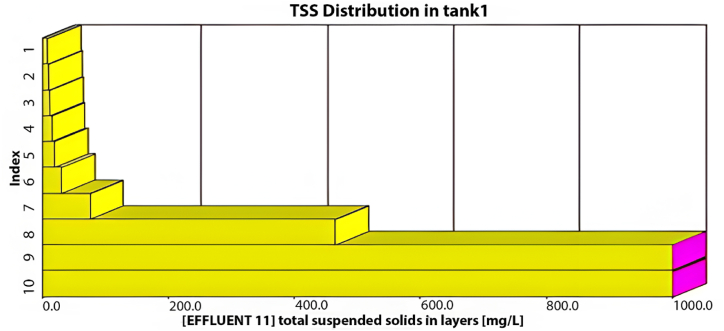
TSS distribution through the layers of the three sedimentation tanks using the proposed layout beyond 35 years.

The simulation results demonstrated the suitability of the previous layout for an additional 10 years, with an expected inflow of around 8800 m^3^/day. Based on the 30-day simulation results, the plant's effluent meets the standards, with TSS removal at 99 %, COD removal at 96 %, and BOD removal at 99.2 %. Additionally, the average DO effluent is 1.57 mg/L. Moreover, the sedimentation tanks produce satisfactory TSS effluent, with the first six layers yielding less than 50 mg/L of TSS, as shown in Fig. 19.

#### 3.3.5 Performance evaluation beyond 40 years

**Fig. 20:**
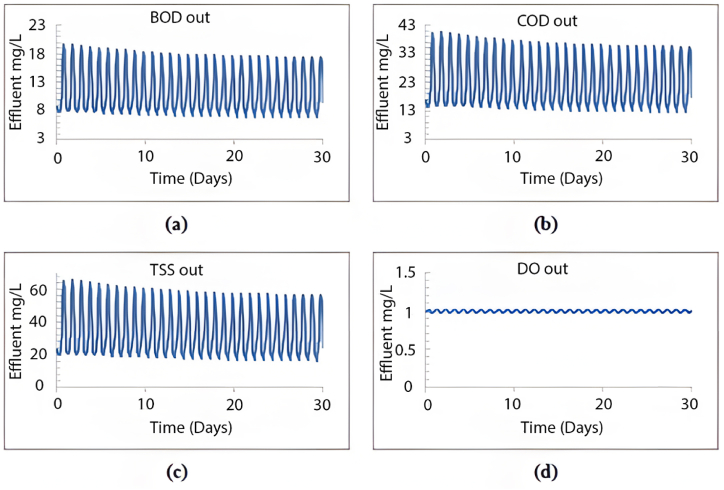
Effluent simulated results for the proposed layout (a) BOD mg/L, (b) COD mg/L, (c) TSS mg/L and (d) DO mg/L beyond 40 years.

After 40 years, the inflow is expected to reach the design inflow rate of 9900 m^3^/day. The GPSX model was used to simulate MWWTP performance, employing the layout used beyond 35 years. Fig. 20 presents the results of effluent TSS, COD, BOD, and DO after a 30-day simulation.

**Fig. 21:**
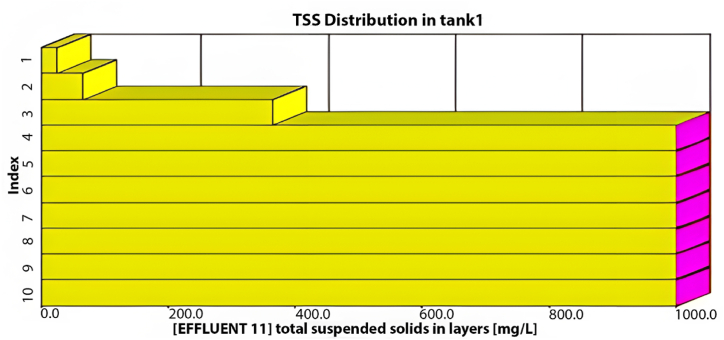
TSS distribution through the layers of the three sedimentation tanks using the proposed layout beyond 40 years.

The removal efficiencies for TSS, COD, and BOD were satisfactory at 98.2 %, 96 %, and 96.8 %, respectively, while the DO effluent decreased to 1.43 mg/L. Additionally, the three sedimentation tanks will be simulated over a 30-day period, as depicted in Fig. 21.

**Fig. 22:**
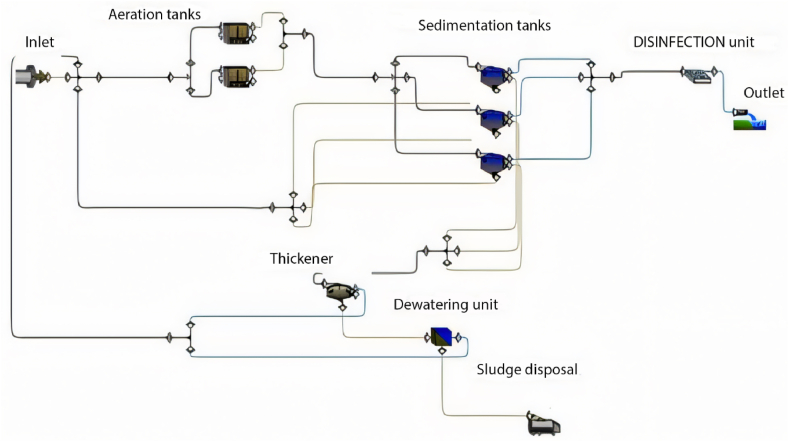
Proposed layout beyond 40 Years.

The simulation results indicate a significant accumulation of sediment in the three sedimentation tanks, resulting in the closure of the tanks. Additionally, it can be observed that the effluent from the first and second layers complies with the specified limits. This outcome suggests that the predicted layout is inadequate to manage the design inflow. Consequently, the current layout requires modification. The proposed adjustment involves operating the second aeration tank in the MWWTP with an equal split fraction alongside the first aeration tank, as illustrated in Fig. 22.

**Fig. 23:**
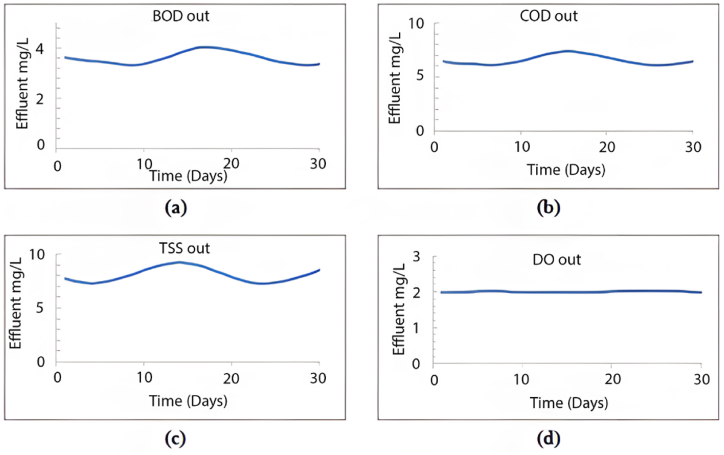
Effluent simulated results for the proposed layout (a) BOD mg/L, (b) COD mg/L, (c) TSS mg/L and (d) DO mg/L beyond 40 years.

The GPS-X model was used to simulate MWWTP performance using the layout with two aeration tanks over a 30-day simulation. The effluent TSS, COD, BOD, and DO are shown in Fig. 23.

**Fig. 24:**
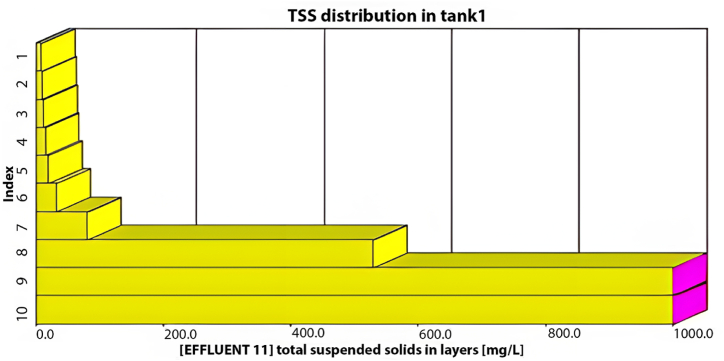
TSS distribution through the layers of the three sedimentation tanks using the new proposed layout beyond 40 years.

The simulation results indicate a TSS removal percentage of 99.1 %, COD removal of 97.4 %, BOD removal of 99.6 %, and an average DO effluent of 2 mg/L. It can be concluded that operating the second aeration tank assists the plant in managing the designed inflow. Furthermore, the simulation results for the three sedimentation tanks illustrate satisfactory performance in the first seven layers, as depicted in Fig. 24. This suggests that the operation of the second aeration tank remains beneficial beyond 40 years with the designed inflow.

**Fig. 25:**
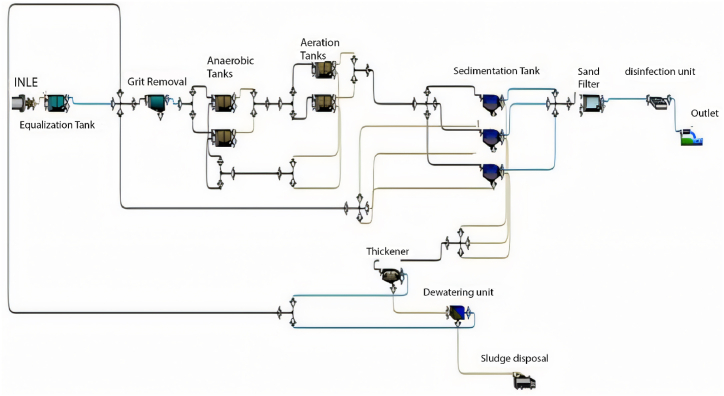
Layout beyond 45 years (full design).

Additionally, the plant was simulated with an inflow exceeding the design capacity. After 45 years, the expected inflow is 11,000 m^3^/day. The full design of the plant will be examined by operating all units and including them in the treatment process. Fig. 25 depicts the layout beyond 45 years (plant's full design).

The simulation results show that the plant's full design is appropriate for an inflow of 11,000 m^3^/day. TSS removal reached 99.8 %, COD removal was 99.6 %, BOD removal was 99.6 %, and the average DO effluent was 2 mg/L. Additionally, the simulation results indicate that the three sedimentation tanks are performing well, particularly the top five effective layers.

#### 3.3.6 Future management strategies for plant expansion

Based on the findings from the previous scenarios, it is evident that the operational lifespan of the plant is limited to 45 years. To extend the plant's functionality beyond this timeframe, we recommend an expansion of the treatment facility. This expansion involves the addition of new treatment units, potentially including sedimentation and aeration tanks. Specifically, our proposal entails integrating a fourth sedimentation tank into the existing infrastructure. However, incorporating a fourth sedimentation tank necessitates the installation of a new thickener unit.

**Fig. 26:**
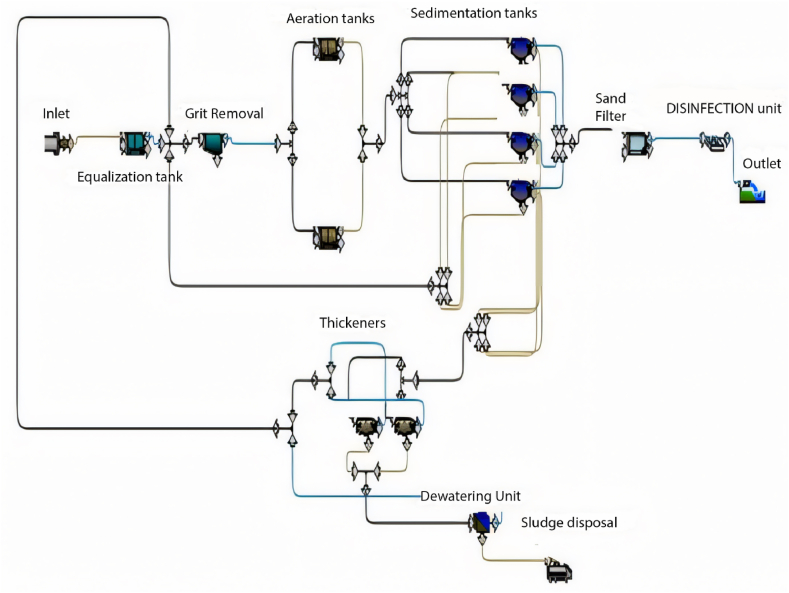
Proposed plant expansion.

Given that the capacity of a single thickener is 750 m^3^/day, and the inflow from one sedimentation tank to the thickener is 250 m^3^/day, the combined inflow from the fourth sedimentation tanks will surpass the capacity of one thickener. Therefore, the addition of a new thickener, coupled with an equal split fraction to the existing one, is proposed to enhance the plant's efficiency over an extended period. Fig. 26 illustrates the proposed layout for the plant's expansion, comprising the inclusion of an extra sedimentation tank and a dedicated thickener unit.

**Fig. 27:**
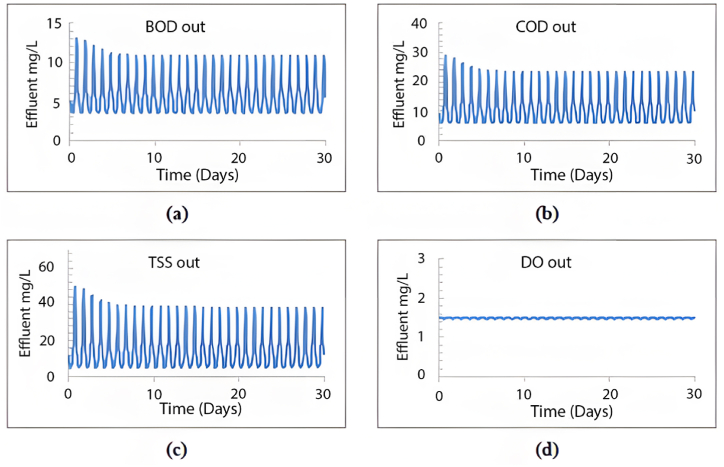
Effluent simulated results using plant's expansion layout beyond 60 years (a) BOD mg/L, (b) COD mg/L, (c) TSS mg/L and (d) DO mg/L.

The GPS-X model was employed to simulate the plant's performance with the expanded layout beyond 50 years, incorporating a fourth sedimentation tank and a second thickener. Effluent parameters such as TSS, COD, BOD, and DO were simulated over a 30-day period at an inflow rate of 12,000 m^3^/day, as depicted in Fig. 27.

The 30-day simulation results indicate that the expanded layout performs effectively with a pumped flow increase to 300 m^3^/day. TSS removal achieved 99.8 %, COD removal reached 99.5 %, BOD removal reached 99.5 %, and the DO effluent was at 1.5 mg/L. Additionally, the distribution of TSS within the sedimentation tank layers meets acceptable standards.

## 4 Discussion

As mentioned in the Results section, numerous and frequent modifications are essential, particularly in the treatment plant design, to ensure optimal efficiency at the lowest cost. Our study, encompassing various scenarios, revealed that population growth significantly influences the number of operational units required, their activation, and the duration of their effectiveness. Population dynamics also significantly impact the physical and chemical characteristics of the flow, necessitating measurement or calculation of various influential factors in plant operation and the maintenance of its efficacy. The region faces environmental challenges, including a scarcity of fresh water, and is frequently affected by external climate events, particularly droughts [6]. This situation necessitates the wise management of water resources [12]. The use of TWW is one of the most promising strategies for conserving and extending available water sources. TWW is regarded as one of the most critical and effective solutions to Jordan's water shortage [14,15]. Previous studies used a GPS-X simulator to improve the performance of WWTPs [23,24]. In Karbala wastewater treatment plants, there was a particular focus on the addition of external carbon sources to enhance denitrification and reduce phosphate concentrations [23], while in the Sharjah case study, the TSS, COD, TKN, and cBOD5 were used to support decision-making strategies and deliver cost savings by reliably evaluating the TWW quality [24] Additionally, some studies used Capdetwork software alongside GPS-X to calculate the economic costs [34]. Our results reveal that the existing layout will not suffice after five years, anticipating an inflow of 4500 m^3^/day. Therefore, the second sedimentation tank needs to operate. Once the plant reaches its maximum design inflow of 10,000 m^3^/day the third sedimentation tank must operate to enhance the plant's performance and sustain it. Previously [23,24], analysed the performance of WWTPs by using the GPS-X simulator and reported an overall performance improvement in the outcomes of the WWTPs and stated the best scenarios. Hence our result is in alignment with previous studies as the majority of them agreed on the beneficial uses of the GPS-X model. Accordingly, The GPS-X software model provided crucial and realistic results, aligning well with the changes observed in the facility due to increased flow volume, primarily driven by population growth. Consequently, the model proves to be an effective and accurate tool, adept at simulating treatment plant realities across a wide spectrum of variables.

## 5 Conclusion

In conclusion, the study has focused on assessing the treatment efficiency of the Al-Marad WWTP through rigorous monitoring and simulation using the GPS-X model. By analysing key parameters such as BOD, COD, TSS, and DO levels in both influent and effluent streams over a period of six months, we have gained valuable insights into the plant's performance dynamics. The GPS-X model has proven to be a powerful tool, accurately simulating current plant performance, and offering strategies for managing potential changes.

The simulations extend beyond short-term assessments, providing projections of the plant's performance over the next 60 years. These projections highlight the need for proactive measures to ensure the plant's long-term viability. Specifically, our findings suggest the importance of modifying the existing layout to accommodate anticipated increases in inflow, as detailed in the study.

Furthermore, our research demonstrates the potential of the GPS-X model in evaluating treatment plant performance in Jordan and beyond. The successful application of the model in simulating the Al-Marad WWTP's performance suggests its reliability for evaluating other treatment plants in the region. Moreover, the model's ability to simulate future operations and assess modifications to the plant's design and operating units underscores its utility as a decision-making tool for administrators and operators.

Looking ahead, we recommend that future researchers explore the integration of machine learning technologies, particularly AI, with GPS-X modelling. By leveraging these advanced technologies, researchers can enhance the accuracy and realism of simulation results, facilitating more informed decision-making in wastewater treatment plant management.

## Data availability statement

Data will be made available on request.

## Additional information

No additional information is available for this paper.

## CRediT authorship contribution statement

**Ayat Sami Odeibat:** Writing – review & editing, Writing – original draft. **Reham Mohammad:** Resources, Project administration, Data curation. **Majed Abu-Zreig:** Supervision.

## Declaration of competing interest

The authors declare that they have no known competing financial interests or personal relationships that could have appeared to influence the work reported in this paper.

## Abbreviations

BOD: Biochemical Oxygen Demand
COD: Chemical Oxygen Demand
STP: Sewage Treatment Plant
TDS: Total Dissolved Solids
TSS: Total Suspended Solids
WRRF: Water Resource Recovery Facility
MWWTP: Al-Marad Wastewater Treatment Plant
WWTP: Wastewater Treatment Plant
TWW: Treated Wastewater
DO: Dissolved Oxygen
TKN: Total Kjeldahl Nitrogen
